# Tear Fluid Biomarkers and Quality of Life in People with Type 2 Diabetes and Dry Eye Disease

**DOI:** 10.3390/metabo13060733

**Published:** 2023-06-08

**Authors:** Mungunshur Byambajav, Andrew Collier, Xinhua Shu, Suzanne Hagan

**Affiliations:** 1 Department of Vision Sciences, School of Health & Life Sciences, Glasgow Caledonian University (GCU), Glasgow G4 0BA, UK; mungunshur.byambajav@gcu.ac.uk; 2 School of Health & Life Sciences, Glasgow Caledonian University (GCU), Glasgow G4 0BA, UK; andrew.collier@nhs.scot; 3 Department of Biological & Biomedical Sciences, School of Health & Life Sciences, Glasgow Caledonian University (GCU), Glasgow G4 0BA, UK; xinhua.shu@gcu.ac.uk

**Keywords:** dry eye disease, type 2 diabetes, tear fluids, biomarkers, inflammatory cytokines, metabolic proteins, multiplex bead analysis, quality of life

## Abstract

Dry eye disease (DED) can be extremely distressing and is common in type 2 diabetes (T2D). To investigate potential biomarkers of DED in T2D, panels of proteins in tears, alongside clinical signs and symptoms of DED, were assessed. Patients were classified into four groups: T2D + DED (*n* = 47), T2D-only (*n* = 41), DED-only (*n* = 17) and healthy controls (*n* = 17). All patients underwent the Ocular Surface Disease Index (OSDI) and Dry Eye-Related Quality of Life (DEQS) questionnaires, tear evaporation rate (TER), fluorescein tear break-up time (fTBUT), corneal fluorescein staining (CFS) and Schirmer 1 test assessments. Six metabolic proteins and 14 inflammatory cytokines were analyzed with multiplex bead analysis. Interleukin (IL)-6 and IL-8 concentrations in tears were significantly higher in the T2D + DED group, and these biomarkers were positively correlated with CFS. In addition, tear IL-6 was negatively correlated with fTBUT in the T2D + DED group. Clinical signs of DED in the T2D + DED group were similar to the DED-only group. The T2D + DED group had more patients with moderate and severe DED (versus the DED-only group), suggesting a different pathogenesis for DED in T2D versus DED-only. Therefore, IL-6 and IL-8 could potentially be diagnostic biomarkers of DED in T2D.

## 1. Introduction

The global prevalence of diabetes in adults was 536.6 million people (10.5%) in 2021 and is increasing at epidemic proportions. It is estimated that there will be 783.2 million people with diabetes (12.2%) by 2045 [1]. As type 2 diabetes (T2D) is a leading systemic risk factor for dry eye disease (DED), the incidence of DED is expected to continue to increase in parallel, and therefore, examination for DED should be an integral part of the ocular examination in patients with T2D [2,3].

DED can lead to serious ocular surface complications, such as punctate keratitis, corneal erosion, corneal scarring, corneal perforation and visual loss [4]. In addition, previous studies have suggested that the effects of DED on the quality of life (QoL) of patients are similar to those observed for patients with angina, hip fractures, or those undergoing dialysis [5,6,7,8]. A recent study has also reported that those with DED showed a greater risk of lower QoL than those with allergic conjunctivitis, glaucoma, macular degeneration, and retinal detachment [9]. Until now, however, very few studies have observed an effect of DED on the QoL of patients with diabetes. A positive association between DED severity and impact on QoL for those with diabetes was observed by Yazdani-ibn-Taz et al. [10] and Hagan et al. [11]. In another study, the authors reported a significantly worse QoL among people with both T2D and DED than that of healthy controls [12].

Due to the multifactorial nature of DED, its diagnosis is challenging for eye care practitioners and tends to differ from practitioner to practitioner [13,14,15,16,17]. The diagnostic algorithm of DED recommended by the Tear Film & Ocular Surface Society, Dry Eye Workshop II (TFOS DEWS, 2017) has not been widely adopted, and practitioners also often underestimate this eye disease [16,18]. More worryingly is the poor repeatability of several clinical tests and a lack of correlation between symptoms and clinical tests [19,20,21]. Thus, many patients are underdiagnosed and/or untreated until they become significantly symptomatic [18]. Various studies have investigated the inflammatory protein (cytokine) profiles of tear fluids from patients with DED as a method of measuring changes to ocular surface health [22,23,24]. Few, however, have assessed cytokines in humans with T2D-associated DED [25]. We have previously shown that metabolic proteins, including Leptin, can be detected in small volumes of healthy tears by bead-based immunoassays [26]. Although, to our knowledge, no published studies have assessed the expression of a panel of metabolic proteins in this patient group (T2D + DED) using this technique.

The purpose of this study was to investigate the role of inflammatory cytokines and metabolic proteins in tear fluids as non-invasive, objective measures of inflammation in the diagnosis of DED among people with T2D. The study compared a panel of biomarkers’ concentrations in tear fluids alongside clinical signs and symptoms of DED in subjects with T2D (with or without DED) and in subjects with DED-only versus healthy controls. The study also investigated how DED affects the QoL of these patient groups. The QoL questionnaire examined the effects that DED had on daily activities, such as reading, watching TV, driving and working. In addition, the study aimed to determine if there were relationships between tear fluid biomarkers’ concentrations and (1) clinical signs and symptoms of DED and (2) clinical data for T2D.

## 2. Materials and Methods

### 2.1. Participants

Participant recruitment and data collection were carried out at the University Hospital Ayr, Ayr, UK, between April 2019 and March 2020. Tear fluid biochemical analysis was performed in the Department of Biological and Biomedical Sciences laboratories, Glasgow Caledonian University (GCU), UK, between February and May 2021. Written informed consent was obtained from all individuals after explaining the study protocol. The study was undertaken in accordance with the principles of the Declaration of Helsinki [27]. The South-Central Hampshire B Research Ethics Committee (no. 18/SC/0509) reviewed and approved the study protocols.

The subjects were classified into four groups: (1) T2D + DED group (subjects with T2D and with DED); (2) T2D-only group (subjects with T2D and without DED); (3) DED-only group (subjects with DED and without T2D); and (4) healthy controls (subjects without T2D and without DED). Subjects with T2D were required to fulfil the criteria defined by the American Diabetes Association [28]. The relevant laboratory results (glycated haemoglobin [HbA_1C_], total cholesterol and high-density lipoprotein [HDL]) were obtained from the medical records of the University Hospital Ayr. DED was diagnosed when at least two of the following criteria were met: (1) DED-related symptom scores of ≥13 with the ocular surface disease index (OSDI) questionnaire; (2) Tear film stability of <10 s by fluorescein tear break-up time (fTBUT) and (3) Tear production of <10 mm in 5 min by the Schirmer 1 test.

The exclusion criteria included: current contact lens wearer, an active ocular allergy, ocular surface inflammation not associated with DED, ocular surgery within the last 12 months, topical ocular therapies, use of artificial tears up to 2 h prior to commencing the study, systemic diseases known to affect tear production, such as Thyroid Eye Disease (Graves’ Disease), Systemic Connective Diseases, and Sjögren’s syndrome.

### 2.2. Clinical Tests

All procedures were undertaken in the right eye and in the same order. The procedures progressed from the least invasive to the most invasive, as follows: (1) OSDI and Dry Eye-related Quality of Life (DEQS) questionnaires; (2) Tear evaporation rate (TER); (3) Tear collection; (4) Schirmer I test; (5) fTBUT and (6) Corneal fluorescein staining (CFS). All procedures were performed over the same time period (between 9 AM and 1 PM; 09:00–13:00) in order to minimize the potential for diurnal variation of biomarkers in tears, TER and tear film stability values and therefore limit their possible impact on the results [22,29,30,31,32,33,34].

#### 2.2.1. OSDI Questionnaire

The OSDI questionnaire (Allergan Inc., Irvine, CA, USA) consists of 12 questions that measure DED symptom severity and its effect on the daily life of the participants over the previous 7 days [5]. It contained three subsections: ocular symptoms, vision-related function and environmental factors. Each item was graded on a scale of 0 “none of the time” to 4 “all of the time”. The final score was calculated with the OSDI formula: the sum of all scores was multiplied by 25 and then divided by the total number of questions answered [5,35]. The final score ranged between 0 and 100, with scores of 0–12 = normal, 13–22 = mild DED, 23–32 =moderate DED and 33–100 = severe DED [5,36].

#### 2.2.2. DEQS Questionnaire

The DEQS questionnaire (Santen Pharmaceutical Co. Ltd. (Osaka, Japan) and Dry Eye Society in Japan) consists of 15 questions and is used to detect the impact of DED on QoL, including the subject’s mental health [10,37,38]. The frequency of the DED symptoms was scored by a 5-point Likert scale, from 0 (no symptoms) to 4 (worst symptoms). The degree of bothersome ocular symptoms was scored by a 4-point Likert scale, from 1 = least symptoms to 4 = worst symptoms [37,39]. When the frequency of symptoms was scored as 0, the degree of bothersome ocular symptoms was also considered as 0. The overall score was calculated by a summary of scores for all of the answered questions and ranged between 0–100, with higher scores indicating greater disability [37,40].

#### 2.2.3. TER Assessment

A non-invasive, commercial and validated instrument, the Eye-Vapometer (Delfin Technologies UK Limited, Surrey, UK), was used to measure TER. To perform TER measurements, subjects were instructed to look at a distant target while seated on a chair. Then, three consecutive TER measurements were obtained with the eyes open, and the mean value was calculated. The subject was allowed to blink at their normal rate during the examination.

#### 2.2.4. Schirmer 1 Test

To evaluate tear production, a sterile Schirmer strip (I-DEW Tearstrips, Entod Re-search Cell UK Ltd., London, UK) was placed in the lower conjunctival sac at the junction of the lateral and middle third. Subjects were asked to close their eyes gently without moving to avoid touching the cornea and in order to reduce reflex tearing. After 5 min, the length of the wetting strip was recorded in millimetres (mm) [41].

#### 2.2.5. fTBUT

Tear film stability was assessed using the fTBUT via a slit-lamp microscope with a cobalt blue filter. The subjects were asked to blink three times following the instillation of fluorescein (Fluoro Fluorescein Sodium Strips, Biotech, UK) and then keep their eyes open. The time was measured in seconds (s) from the last blink to the appearance of dark spots or lines in the fluorescein-stained tear film. Three measurements were taken, and the mean value was calculated. Between the measurements, subjects were allowed to blink normally.

#### 2.2.6. CFS

Immediately after the fTBUT evaluation, corneal integrity was examined by CFS. The study employed the Oxford Grading Scale, which uses a chart to indicate the amount of ocular surface staining labelled in order of increasing severity (0-Normal, I-Trace, II-Mild, III-Moderate, IV-Severe). The examiner chose the grade that best matched their view of the corneal surface [42].

### 2.3. Tear Collection and Tear Fluid Analysis

A total of 2 microliters (µL) of unstimulated basal tears were collected from each subject in a maximum of 10 min (1 µL for metabolic protein analysis, 1 µL for inflammatory cytokine analysis). Tears (1 µL) were taken from subjects using sterile, disposable, glass capillary micropipettes (“microcaps”, Drummond, Broomall, PA, USA) from the lateral canthus of the subjects’ open eyes to minimize contact with the ocular surface and thus minimize reflex tearing. The subjects were allowed to blink normally during the tear collection. The collected tears were expelled immediately from the micropipette into a 0.5 mL lo-bind sterile Eppendorf tube (Sigma, Gillingham, Dorset, UK) and diluted 1:10 in 9 μL of assay buffer (Merck Millipore, Watford, UK). This low volume of 1 µL tears has been previously shown to be sufficient for cytokine analysis when performing a low-volume protocol, which uses only 10 μL volumes of samples and standards instead of the 25–50 μL used in regular protocols [23,43,44,45]. The diluted tears were kept on ice for no more 2 h and then centrifuged at 14,000 rpm for 1 min at 4 °C before transferring to a −80 °C freezer.

A panel of six metabolic proteins (Leptin; Insulin; Glucagon; total Glucagon-Like Peptide [GLP]-1; active Ghrelin and C-peptide) was analyzed using the Human Metabolic Hormone Magnetic Bead Panel (Milliplex, Merck Millipore, UK). The concentrations of 14 inflammatory cytokines (Epidermal Growth Factor [EGF]; Fractalkine; Interferon [IFN]-γ; Interleukin-1 receptor antagonist-1RA [IL-1RA]; IL-10; IL-1β; IL-2; IL-4; IL-6; IL-8; IFN-γ inducible protein (IP)-10; Monocyte Chemoattractant Protein [MCP]-1; Tumour Necrosis Factor (TNF)-α and Vascular Endothelial Growth Factor [VEGF]) were analyzed using the Human Cytokine/Chemokine/Growth Factor Panel A Magnetic Bead Panel (Milliplex, Merck Millipore, UK). Final protein concentrations were measured using Bio-Plex software (Bio-Plex Manager 6.1 Software, Bio-Rad, Hercules, CA, USA) in a Luminex 200 machine (Luminex Corp, Austin, TX, USA). The reason these metabolic proteins were specifically chosen was that most of their actions could contribute to the metabolic syndrome, which is important in the aetiopathogenesis of T2D. In addition, previous work in our group has shown that Leptin, Insulin, active Ghrelin and C-peptide are detectable in normal human tears [26].

The samples were analyzed following the manufacturer’s protocol. The minimum detectable concentrations (MinDC in pg/mL) were Leptin = 41, Insulin = 87, Glucagon = 13, total GLP-1 = 2.5, active Ghrelin = 13, C-peptide = 9.5, EGF = 3.2, Fractalkine = 29.75, IFN-γ = 0.86, IL-10 = 0.91, IL-1RA = 1.29, IL-1β = 0.52, IL-2 = 0.28, IL-4 = 0.2, IL-6 = 0.14, IL-8 = 0.52, IP-10 = 2.13, MCP-1 = 3.05, TNF-α = 5.39 and VEGF = 0.98.

For tear fluid biomarker analysis, some biomarker concentrations were marked by the analysis as “Out of Range” (<OOR), meaning the value was less than the MinDC. Alternatively, the values were extrapolated beyond the standard range, meaning that the values were outside the standard curve range. We assigned a value for <OOR as equal to the midpoint between the MinDC and zero [46]. There are various ways to select the value for analysis when the response is below the detection limit. Assigning zero for <OOR reduces the power of the analysis and biases the data downward, suggesting no data are available when there is information available. While using the minimum detection limit for <OOR biases estimates upward. Therefore, choosing a mid-point between 0 and the minimum detection limit is likely to average out the bias for OOR data. This method of assigning OOR values by constraining them to between zero and the detection limit of the analyte has been described previously, including a recent comparison of multiplex cytokine assays [46,47]. The extrapolated values were accepted as true values. To avoid biased results, statistical analysis was restricted to proteins with a percentage of detection values of 50% or higher, i.e., with <50% of samples falling below the OOR. Molecules detected in less than 50% of the samples were not statistically analyzed any further [31,48,49,50].

### 2.4. Statistical Analysis

Statistical analysis was performed using SPSS statistical software (Statistical Package for the Social Sciences, Version 26, Armonk, NY, USA). Descriptive parameters were expressed as the median (Interquartile range (IQR)) based on the normality assessed using the Shapiro–Wilk test. Differences in sex among groups were tested using the Chi-Square test. The Kruskal–Wallis test was used to compare the demographics, clinical signs and symptoms of DED and tear fluid biomarkers’ concentrations between the study groups. As the data were not normally distributed, the Mann–Whitney U test was used to compare the duration of T2D, HbA_1C_, total Cholesterol and HDL between the T2D-only and T2D + DED groups.

Relationships between the variables were calculated using the Spearman correlation test. The Spearman correlation coefficient (r_s_) was used to determine if relationships existed. Due to the different numbers of subjects in each group, an r_s_ of >0.46 was considered a positive relationship, and <−0.46 was considered a negative relationship in the healthy controls and DED-only groups. An r_s_ of >0.31 was considered a positive relationship, and <−0.31 was considered a negative relationship in the T2D-only group. An r_s_ of >0.27 was considered a positive relationship, and <−0.27 was considered a negative relationship in the T2D + DED group [47,51]. A *p* value of ≤0.05 was considered statistically significant.

## 3. Results

### 3.1. Demographics, Clinical Signs and Symptoms of DED and Clinical Data of T2D

In total, 122 subjects were enrolled, with *n* = 17 in the healthy controls, *n* = 17 in the DED-only group, *n* = 41 in the T2D-only group and *n* = 47 in the T2D + DED group. The demographics, clinical signs and symptoms of DED and clinical data of T2D in each group are summarized in Table 1. Comparisons (*p*-values) of these characteristics are detailed in Table 2.

The sex of each group was matched (*p* = 0.14, Table 1). The patients in the T2D + DED group were older than the healthy controls and the patients in the DED-only group (*p* = 0.001 and *p* = 0.03, respectively, Table 2). In addition, the patients in the T2D-only group were older than the healthy controls (*p* = 0.003, Table 2). No differences were found for the duration of T2D, HbA_1C_, total Cholesterol and HDL levels between the T2D-only and T2D + DED groups (*p* = 0.21, *p* = 0.45, *p* = 0.45 and *p* = 0.28, respectively; Table 1).

For TER, there were no differences between the four groups (*p* = 0.44, Table 1). The results of the other comparison tests, however, showed that the fTBUT, CFS, Schirmer 1 values, OSDI and DEQS scores were significantly different across the study groups (*p* < 0.001, *p* = 0.002, *p* < 0.001, *p* < 0.001 and *p* < 0.001, respectively; Table 1). In more detail, the T2D + DED and the DED-only groups had significantly *lower* fTBUT values compared to the healthy controls (*p* = 0.004 and *p* = 0.01, respectively, Table 2) and the T2D-only group (*p* < 0.001 and *p* = 0.003, respectively). In addition, the T2D + DED and DED groups showed significantly *lower* Schirmer 1 values than the healthy controls (*p* < 0.001) and the T2D-only group (*p* < 0.001). The CFS of the patients in the T2D + DED group was significantly *higher* than the T2D-only group (*p* = 0.003, Table 2) and the healthy controls (*p* = 0.001). In the T2D + DED and DED groups, the OSDI scores were significantly *higher* than in healthy controls (*p* < 0.001 and *p* = 0.001, respectively) and the T2D-only group (*p* < 0.001 and *p* = 0.002, respectively). Moreover, the DEQS scores in the T2D + DED and DED groups were significantly *higher* versus the healthy controls (*p* = 0.001 and *p* = 0.01, respectively, Table 2) and T2D-only group (*p* < 0.001 and *p* = 0.002, respectively). No differences were detected for the fTBUT, CFS, Schirmer 1 test, OSDI and DEQS scores between the T2D + DED and DED groups (*p* > 0.05). In addition, the fTBUT, CFS, Schirmer 1 test, OSDI and DEQS scores of patients in the T2D-only group were similar to healthy controls (*p* > 0.05).

The patients with DED were classified into subgroups as patients with mild DED, patients with moderate DED and patients with severe DED, according to their OSDI questionnaire results. Mild DED was considered when patients had OSDI scores of 13–22, and moderate DED was considered when patients had OSDI scores of 23–32. In addition, severe DED was considered when patients had OSDI scores of 33–100. Asymptomatic DED was also diagnosed when patients had OSDI scores of <13, but they had both fTBUT of <10 s and Schirmer 1 test values of <10 mm. Among the subjects in the DED-only group, six had mild (35.3%), one had moderate (5.9%) and four had severe DED (23.5%), as assessed using the above criteria. In addition, six patients were asymptomatic (35.3%) in the DED-only group. Among patients in the T2D + DED group, fifteen were asymptomatic (31.9%), twelve had mild (25.5%), eight had moderate (17%) and twelve had severe DED (25.6%).

### 3.2. Biomarkers’ Concentrations in Tears among Groups

The detection values of two metabolic proteins out of six were found in more than 50% of the tear samples (Insulin and Leptin). In addition, eleven cytokines out of 14 were found in more than 50% of the samples analyzed (IL-1RA, IL-6, IL-8, EGF, Fractalkine, IL-1β, IL-10, IP-10, MCP-1, TNF-α and VEGF). The metabolic proteins of Glucagon, total GLP-1, active Ghrelin and C-peptide, and the inflammatory cytokines of IL-2, IL-4 and IFN-γ were not further analyzed, as they were detected in less than 50% of the samples. The concentrations of biomarkers in each group are detailed in Table 3. Comparison test results (*p*-values) of biomarkers between the four groups are detailed in Table 4.

The concentrations of IL-1RA, IL-6 and IL-8 were found to be significantly different across the study groups (*p* = 0.01, *p* = 0.005 and *p* = 0.03; Table 3). For example, there were significantly *lower* concentrations of IL-RA in the T2D-only group versus the T2D + DED group and versus the healthy controls (*p* = 0.002 and *p* = 0.05, respectively, Table 4). The T2D + DED group showed significantly *higher* concentrations of IL-6 in tears compared to healthy controls, the DED-only group and the T2D-only group (*p* = 0.02, *p* = 0.001 and *p* = 0.03, respectively, Table 4). Tear IL-8 concentrations were found to be significantly *higher* in the T2D + DED group versus the healthy controls, the DED-only group and the T2D-only group (*p* = 0.01, *p* = 0.05 and *p* = 0.03, respectively, Table 4). No differences were found for EGF, Fractalkine, IL-1β, IL-10, IP-10, MCP-1, TNF-α, VEGF, Insulin and Leptin concentrations when comparing the groups (*p* > 0.05; Table 3).

### 3.3. The Relationship between Tear Fluid Biomarkers and Clinical Signs and Symptoms of DED

The relationships between the biomarkers and clinical signs of DED were calculated for the DED-only group and for the T2D + DED group. The significance levels of these variables and their clinical interpretation in the DED-only and T2D + DED groups are detailed in Supplementary Materials (Table S1 and Table S2, respectively). In addition, the relationships between the biomarkers and symptoms of DED were calculated for the DED-only group and for the T2D + DED group. The significance levels of the variables and their clinical interpretation in the DED-only and T2D + DED groups are detailed in Supplementary Materials (Table S3 and Table S4, respectively).

Negative relationships were found for EGF versus TER (r_s_ = −0.50, *p* = 0.05), IL-6 versus Schirmer 1 values (r_s_ = −0.5, *p* = 0.05) and Leptin versus Schirmer 1 values (r_s_ = −0.5, *p* = 0.03) in the DED-only group (Table S1). Positive relationships were shown for IL-6 versus TER (r_s_ = 0.6, *p* = 0.02) and for Leptin versus TER (r_s_ = 0.5, *p* = 0.02) in the DED-only group (Table S1). In the T2D + DED group, there were negative relationships between IL-10 versus TER (r_s_ = −0.3, *p* = 0.05; Table S2), IL-1β versus TER (r_s_ = −0.3, *p* = 0.02), IL-1RA versus fTBUT (r_s_ = −0.3, *p* = 0.04), IL-1RA versus Schirmer 1 values (r_s_ = −0.5, *p* < 0.001), IL-6 versus fTBUT (r_s_ = −0.3, *p* = 0.05) and MCP-1 versus Schirmer 1 values (r_s_ = −0.3, *p* = 0.03). In addition, CFS among patients in the T2D + DED group was positively correlated with IL-8 (r_s_ = 0.3, *p* = 0.04), IL-6 (r_s_ = 0.3, *p* = 0.02) and MCP-1 concentrations in tears (r_s_ = 0.3, *p* = 0.03; Table S2).

In the DED-only group, positive relationships were found between DEQS and IP-10 (r_s_ = 0.57, *p* = 0.02, Table S3) TNF-α (r_s_ = 0.49, *p* = 0.04) and VEGF concentrations (r_s_ = 0.49, *p* = 0.05). In addition, a positive relationship was detected between VEGF concentrations and OSDI scores for the DED-only group (r_s_ = 0.47, *p* = 0.06, Table S3). There were no relationships detected between the DEQS scores and biomarkers in the T2D + DED group (Table S4). Moreover, no relationships existed between the biomarkers and OSDI scores in the T2D + DED group (Table S4).

### 3.4. The Relationship between Tear Fluid Biomarkers and Clinical Data of T2D

The relationships between the biomarkers and clinical data of diabetes were calculated for the T2D + DED group. The significance levels of the variables and their clinical interpretation are detailed in Supplementary Materials (Table S5).

The duration of T2D negatively correlated with tear fluid VEGF (r_s_ = −0.3, *p* = 0.04; Table S5) and Insulin concentrations (r_s_ = −0.3, *p* = 0.03). In addition, tear Insulin concentrations were negatively correlated with HDL levels (r_s_ = −0.39, *p* = 0.007).

### 3.5. An Effect of DED on QoL

**Figure 1:**
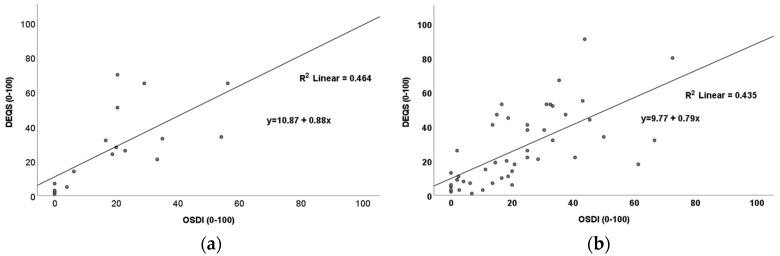
Scatter plots showing positive relationships between OSDI and DEQS for (**a**) the DED–only group (*p* < 0.001; r_s_ = 0.79) and (**b**) the T2D + DED group (*p* < 0.001; r*_s_* = 0.73).

A determination of the relationship between DED severity and QoL was undertaken to detect if there was an effect of DED-related symptoms on patient QoL. It was noted that there was a significant positive relationship between DED severity (as measured by OSDI) and impact on QoL (as measured by DEQS) in the DED-only group (*p* < 0.001, r_s_ = 0.79; Figure 1a) and the T2D + DED group (*p* < 0.001, r_s_ = 0.73; Figure 1b).

## 4. Discussion

This study was performed to identify potential tear fluid biomarkers in T2D-related DED and compare them to the established clinical and laboratory measures used in DED diagnosis. To the best of our knowledge, the only documented data available to evaluate tear fluid IL-10, IP-10, Fractalkine, IL-1RA, IL-8, IL-6, VEGF, MCP-1, Insulin and Leptin concentrations in patients with both DED and T2D was by our previous study [52]. The current study showed significantly *higher* tear concentrations of IL-6 and IL-8 in the T2D + DED group than the other three groups, and these two biomarkers positively correlated with CFS in this study group. Furthermore, we found that tear fluid IL-6 concentrations were negatively correlated with fTBUT in the T2D + DED group. We previously reported data on IL-6 and IL-8 in tears of patients with both T2D and DED in 2020 [52]. This previous research was a pilot study conducted in a small cohort with 21 patients. The pilot study detected the *highest* concentrations of IL-6 in tears of the T2D + DED group, and the biomarker was not associated with any of the DED clinical data in this study group [52]. In a study by Wu et al. [53], tear fluid IL-6 and IL-8 concentrations were significantly higher in an MGD-related DED group than in the healthy controls, and these biomarkers were negatively correlated with the Schirmer 1 test (Schirmer 1 test versus IL-8, r_s_ = −0.37, *p* = 0.01; Schirmer versus IL-6, r_s_ = −0.38, *p* = 0.001). In another recent study, tear fluid IL-8 concentrations were significantly higher in patients with diabetes than for healthy controls, and IL-8 concentrations were higher in patients with more advanced stages of diabetes [54]. IL-6 and IL-8 are well-known pro-inflammatory cytokines that play an important role in mediating inflammation systemically, whereas on the ocular surface, they are secreted from damaged epithelial cells and induce various signs of ocular surface stress [55]. The current study showed that the CFS scores were *highest* in the T2D + DED group, which might be attributable to the increased tear concentrations of IL-6 and IL-8, as well as the increased number of patients with moderate and severe DED in this group.

A panel of diagnostic indicators was used to investigate the clinical signs and symptoms of DED among people with T2D and DED. These were compared to individuals with T2D-only, DED-only and healthy controls. Overall, we showed that the clinical signs and symptoms of DED were similar for patients in the T2D-only group and the healthy controls. In addition, patients in the T2D + DED group had similar clinical signs and symptoms to patients in the DED-only group. This is comparable to previous studies [25,53,56,57]. For example, a recent study investigated the characteristics of the Meibomian glands among patients with T2D and reported that DED in T2D had similar DED severity and tear film quality to DED in non-T2D patients [53]. OSDI, TBUT and Schirmer values were previously found to be similar among patients in the T2D + DED group and the DED-only group [25,56,57]. In the current study, however, the DED-only group had more patients with *mild* DED (versus the T2D + DED group), while the T2D + DED group had more patients with *moderate and severe* DED (versus the DED-only group). These findings are similar to those reported previously by Manchikanti et al. [58]. This may suggest a different pathogenesis of DED severity in T2D versus DED alone.

A secondary aim was to investigate the effect of DED severity on the QoL of patients. The present study observed a similar QoL of patients in the T2D + DED group with patients in the DED-only group. The QoL of patients in the T2D + DED group, however, was significantly worse than in the T2D-only group and healthy controls. Our study also demonstrated a positive association between DED severity and its effect on QoL in both the T2D + DED and DED-only groups, which was comparable to previous studies [10,11].

There were some limitations in this study. The first one is an unequal number of patients were recruited in each study group, which could reduce the ability to detect true differences. In addition, there were differences in the median age between the study groups. Unfortunately, due to the onset of the COVID-19 pandemic, we could not recruit a larger number of patients. Therefore, further studies in an equal number of subjects and other age groups are warranted. Another limitation is that the study did not ask for the general medical history, dietary and fasting status of the participants. It may be important for future studies to determine metabolic proteins in pre- and post-prandial tears in people with both DED and T2D, as these proteins are affected by eating.

Ageing is a risk for developing DED in the general population [59], and DED remains a common problem, particularly in individuals with T2D [60]. However, as the mean age that diabetes occurs is decreasing globally, there is a corresponding increase in DED incidence in younger generations and children [61,62]. Moreover, a study by Zou et al. [57] reported that the pathogenesis of diabetes-associated DED was very similar in adults and children, with differentially expressed tear proteins of adults and children with T2D being associated with inflammation, immune factors, and lipid metabolism.

DED remains a common problem, particularly in individuals with T2D. DED can often negatively impact QoL much in the same way that angina, hip replacements and renal replacement therapy can [5,6,7,8]. These patients often have many other problems and co-morbidities, such as poor glycaemic control, hypertension and dyslipidaemia, plus macro- and microvascular complications. The increase in DED in T2D is commonly not recognized by diabetologists. DED is, therefore, often overlooked by clinicians. If a simple tear or blood test were available to detect DED more easily, DED would be managed more appropriately. This study has investigated potential biomarkers of DED and, in many respects, has only “scratched the surface”. We believe that this research has provided a good platform for others to base their research and go on to determine a possible marker for DED.

## 5. Conclusions

Inflammation is important in the cause and progression of DED, with concomitant cytokine production occurring. Various studies have shown that inflammatory cytokines, including IL-6 and IL-8, have a role in the pathogenesis of DED. Thus, cytokines may be used to measure changes in ocular surface health. In this study, tear fluid IL-6 and IL-8 concentrations correlated with various clinical signs of dry eye in T2D with DED and could potentially be diagnostic biomarkers of T2D-related DED. Clinical signs of DED in the T2D + DED group were similar to the DED-only group. The T2D + DED group, however, had more patients with moderate and severe DED (versus the DED-only group), suggesting a different pathogenesis for T2D-associated DED versus DED-alone.

## Figures and Tables

**Table 1:** Demographics, clinical signs and symptoms of DED and clinical data of T2D among groups. Data are expressed as Median (IQR).

Parameters	Healthy Control (*n* = 17)	DED-Only (*n* = 17)	T2D-Only (*n* = 41)	T2D + DED (*n* = 47)	*p*-Values
Sex					0.14
M, *n* (%)	6 (35.3%)	7 (41.2%)	25 (61%)	29 (61.7%)	
F, *n* (%)	11 (64.7%)	10 (58.8%)	16 (39%)	18 (38.3%)	
Age (years)	52 (16) ^c,d^	60 (20) ^d^	62 (21) ^a^	64 (16) ^a,b^	*0.03*
T2D duration (years)	N/A	N/A	10.6 (8.7)	15.4 (9.4)	0.21
TER (g/m^2^h)	46.3 (18)	48 (30)	51.6 (26.7)	48.6 (33.6)	0.48
fTBUT (s)	7 (5.5) ^b,d^	4 (4.5) ^a,c^	8 (5.5) ^b,d^	5 (2) ^a,c^	<*0.001*
CFS (0–4)	0 (0) ^d^	0 (1)	0 (0) ^d^	1 (1) ^a,c^	*0.002*
Schirmer (mm)	20 (23) ^b,d^	8 (12) ^a,c^	18 (12) ^b,d^	8 (14) ^a,c^	<*0.001*
OSDI (0–100)	2 (3.2) ^b,d^	20 (29.2) ^a,c^	2.2 (6.9) ^b,d^	18.7 (27.1) ^a,c^	<*0.001*
DEQS (0–100)	8 (8.5) ^b,d^	26 (36.5) ^a,c^	6 (8.5) ^b,d^	21 (35) ^a,c^	<*0.001*
HbA_1C_ (mmol/L)	N/A	N/A	64 (23.5)	60 (25)	0.45
Total Cholesterol (mmol/L)	N/A	N/A	4.1 (1.4)	3.8 (1.9)	0.45
HDL (mmol/L)	N/A	N/A	1.25 (0.4)	1.18 (0.51)	0.28

DED, Dry Eye Disease; T2D, type 2 diabetes; N/A, not applicable; M, male; F, female; OSDI, ocular surface disease index; DEQS, dry eye-related quality of life; TER, tear evaporation rate; fTBUT, fluorescein tear break-up time; CFS, corneal fluorescein staining; HbA_1C_, glycated haemoglobin; HDL, high-density lipoprotein; n, the number of subjects. Note: a *p* value of ≤0.05 was considered a statistically significant difference (shown in *italics*). ^a^ *p* ≤ 0.05 vs. healthy control group; ^b^ *p* ≤ 0.05 vs. DED-only group; ^c^ *p* ≤ 0.05 vs. T2D-only group; ^d^ *p* ≤ 0.05 vs. T2D + DED group.

**Table 2:** Comparison (*p*-values) of demographics, clinical signs and symptoms of DED among groups.

Parameters	T2D + DED vs. DED-Only	T2D + DED vs. T2D-Only	T2D + DED vs. Healthy Controls	T2D-Only vs. DED-Only	T2D-Only vs. Healthy Controls	DED-Only vs. Healthy Controls
Age (years)	*0.03*	0.81	*0.001*	0.06	*0.003*	0.37
fTBUT (s)	0.84	<*0.001*	*0.004*	*0.003*	0.93	*0.01*
CFS (0–4)	0.19	*0.003*	*0.001*	0.35	0.24	0.08
Schirmer (mm)	0.63	<*0.001*	<*0.001*	<*0.001*	0.55	<*0.001*
OSDI (0–100)	0.53	<*0.001*	<*0.001*	*0.002*	0.29	*0.001*
DEQS (0–100)	0.91	<*0.001*	*0.001*	*0.002*	0.89	0.01

Note: a *p* value of ≤0.05 was considered a statistically significant difference (shown in *italics*).

**Table 3:** Concentrations of biomarkers (pg/mL) among groups. Data are expressed as Median (IQR).

Biomarkers	Healthy Controls (*n* = 17)	DED-Only (*n* = 17)	T2D-Only (*n* = 41)	T2D + DED (*n* = 47)	*p*-Values
IL-1RA	4111.5 (7679.6) ^c^	2080 (11,699.6)	869 (2969.6) ^a,d^	3159.6 (9877.7) ^c^	*0.01*
IL-6	3.3 (40.5) ^d^	0.7 (17.6) ^d^	5.6 (61.9) ^d^	28.3 (85.6) ^a,b,c^	*0.005*
IL-8	94.9 (103.5) ^d^	141.1 (287.9) ^d^	163.2 (278) ^d^	279.5 (645.5) ^a,b,c^	*0.03*
EGF	1817.9 (1137.6)	1586.1 (1028)	1324.9 (1158)	1634.5 (831.1)	0.45
Fractalkine	1182 (648.7)	1135.1 (921)	1084 (377)	1052.5 (410.5)	0.9
IL-1β	10.9 (47.9)	36.5 (57.9)	24.4 (51.2)	24.4 (56)	0.64
IL-10	27.4 (74.2)	41 (85.1)	27.4 (61.9)	27.4 (96.9)	0.8
IP-10	15,541.5 (20,968)	31,954 (31,916)	19,541 (24,780)	21,055 (34,857)	0.26
MCP-1	257.5 (1363.4)	900.7 (1296.2)	394 (820.2)	720.1 (1305.4)	0.06
TNF-α	27 (40.7)	27.5 (45.5)	36.7 (29.3)	39.9 (26.4)	0.82
VEGF	629 (479.8)	423.9 (456.8)	488.3 (346.1)	553.1 (477.6)	0.5
Insulin	435 (645.2)	517.6 (1032.7)	821.2 (1083.8)	1203.4 (2278.5)	0.2
Leptin	73.1 (24.6)	74.8 (40.2)	73.1 (22.7)	77.7 (23.1)	0.75

DED, Dry Eye Disease; T2D, type 2 diabetes. Note: a *p* value of ≤0.05 was considered a statistically significant difference (shown in *italics*). ^a^ *p* ≤ 0.05 vs. healthy control group; ^b^ *p* ≤ 0.05 vs. DED-only group; ^c^ *p* ≤ 0.05 vs. T2D-only group; ^d^ *p* ≤ 0.05 vs. T2D + DED group.

**Table 4:** Comparison (*p*-values) of biomarkers of DED among groups.

Parameters	T2D + DED vs. DED-Only	T2D + DED vs. T2D-Only	T2D + DED vs. Healthy Controls	T2D-Only vs. DED-Only	T2D-Only vs. Healthy Controls	DED-Only vs. Healthy Controls
IL-1RA	0.61	*0.002*	0.68	0.07	*0.05*	0.93
IL-6	*0.001*	*0.03*	*0.02*	0.12	0.54	0.44
IL-8	*0.05*	*0.03*	*0.01*	0.77	0.42	0.66

Note: a *p* value of ≤0.05 was considered a statistically significant difference (shown in *italics*).

## Data Availability

The data presented in this study are available on request from the corresponding author. The data are not publicly available due to privacy.

## Supplementary Materials

The following supporting information can be downloaded at: https://www.mdpi.com/article/10.3390/metabo13060733/s1, Table S1: Relationships of biomarkers and ocular surface parameters in the DED-only group. R*_s_* and *p* values are shown, as well as the clinical interpretation of these correlations. A *p* value of ≤0.05 was considered statistically significant. Due to there being a total of 17 subjects in the DED-only group, an r*_s_* of >0.46 is considered a positive correlation and <−0.46 is considered a negative correlation; Table S2: Relationships of biomarkers and ocular surface parameters in the T2D + DED group. R*_s_* and *p* values are shown, as well as the clinical interpretation of these relationships. A *p* value of ≤0.05 is considered as statistically significant. Due to there being a total of 47 subjects in the T2D + DED group, an r*_s_* of >0.27 is considered a positive relationship and <−0.27 is considered a negative relationship; Table S3: Relationships of biomarkers and symptoms of DED in the DED-only group. R*_s_* and *p* values are shown, as well as the clinical interpretation of these correlations. A *p* value of ≤0.05 was considered statistically significant. Due to there being a total of 17 subjects in the DED-only group, an r*_s_* of >0.46 is considered a positive correlation and <−0.46 is considered a negative correlation; Table S4: Relationships of biomarkers and symptoms of DED in the T2D + DED group. R*_s_* and *p* values are shown, as well as the clinical interpretation of these relationships. A *p* value of ≤0.05 is considered as statistically significant. Due to there being a total of 47 subjects in the T2D + DED group, an r*_s_* of >0.27 is considered a positive relationship and <−0.27 is considered a negative relationship; Table S5: Relationships of biomarkers and clinical data of T2D in the T2D + DED group. R*_s_* and *p* values are shown, as well as the clinical interpretation of these relationships. A *p* value of ≤0.05 is considered as statistically significant. Due to there being a total of 47 subjects in the T2D + DED group, an r*_s_* of >0.27 is considered as a positive relationship and <−0.27 is considered as a negative relationship.
