# Supplementary material for: Tear Fluid Biomarkers and Quality of Life in People with Type 2 Diabetes and Dry Eye Disease
Source: Metabolites. 2023 Jun 8;13(6):733. doi: 10.3390/metabo13060733 (PMC10305240; doi:10.3390/metabo13060733)
Supplement: Supplementary file 1 [file metabolites-13-00733-s001.zip › metabolites-2407509-supplementary.pdf]

Table S1. Relationships of biomarkers and ocular surface parameters in the DED-only group. R_s and *p* values are shown, as well as the clinical interpretation of these correlations. A *p* value of ≤0.05 was considered statistically significant. Due to there being a total of 17 subjects in the DED-only group, an r_s of > 0.46 is considered as a positive correlation and <-0.46 is considered as a negative correlation [47, 48].

**Biomarkers**	**TER**			**fTBUT**			**CFS**			**Schirmer 1**		
	r_s	***p*-value**	**Interpretation**	r_s	***p*-value**	**Interpretation**	r_s	***p*-value**	**Interpretation**	r_s	***p*-value**	**Interpretation**
IL-1RA	-0.1	0.7	N/A	-0.4	0.1	N/A	0.1	0.6	N/A	-0.2	0.4	N/A
IL-8	0.2	0.5	N/A	-0.02	0.9	N/A	0.1	0.6	N/A	-0.05	0.8	N/A
EGF	*-0.5*	*0.05*	*↑TER ↓EGF*	0.1	0.6	N/A	-0.4	0.2	N/A	-0.03	0.9	N/A
Fractalkine	0.1	0.7	N/A	-0.2	0.4	N/A	0.2	0.5	N/A	0.02	0.9	N/A
IL-10	0.08	0.8	N/A	0.1	0.6	N/A	-0.02	0.9	N/A	0.08	0.7	N/A
IL-1β	0.3	0.3	N/A	-0.008	0.9	N/A	0.07	0.8	N/A	0.09	0.7	N/A
IL-6	*0.6*	*0.02*	*↑TER ↑IL-6*	-0.02	0.9	N/A	-0.008	0.9	N/A	*-0.5*	*0.05*	*↓Schirmer ↑IL-6*
IP-10	0.09	0.7	N/A	-0.2	0.4	N/A	0.3	0.2	N/A	-0.1	0.6	N/A
MCP-1	-0.4	0.2	N/A	-0.2	0.5	N/A	0.09	0.7	N/A	0.08	0.8	N/A
TNF-α	0.02	0.9	N/A	-0.2	0.4	N/A	0.1	0.6	N/A	-0.3	0.2	N/A
VEGF	-0.4	0.09	N/A	0.3	0.3	N/A	-0.2	0.5	N/A	0.2	0.5	N/A
Leptin	*0.5*	*0.02*	*↑TER ↑Leptin*	-0.1	0.7	N/A	0.2	0.3	N/A	*-0.5*	*0.03*	*↓Schirmer ↑Leptin*
Insulin	-0.1	0.7	N/A	-0.3	0.3	N/A	0.04	0.9	N/A	-0.4	0.2	N/A

Key words: TER, tear evaporation rate; fTBUT, fluorescein tear break-up time; CFS, corneal fluorescein staining; r_s, Spearman correlation coefficient; N/A, not applicable.

Note: Statistically significant differences and their clinical interpretation are reported in *italics*.

Table S2. Relationships of biomarkers and ocular surface parameters in the T2D+DED group. R_s and *p* values are shown, as well as the clinical interpretation of these relationships. A *p* value of ≤0.05 is considered as statistically significant. Due to there being a total of 47 subjects in the T2D+DED group, an r_s of > 0.27 is considered as a positive relationship and < -0.27 is considered as a negative relationship [47, 48].

Biomarkers	TER			fTBUT			CFS			Schirmer 1 test		
	r_s	*p*-value	Interpretation	r_s	*p*-value	Interpretation	r_s	*p*-value	Interpretation	r_s	*p*-value	Interpretation
IL-1RA	-0.1	0.4	N/A	*-0.3*	*0.04*	*↓fTBUT ↑IL-1RA*	0.2	0.1	N/A	*-0.5*	*<0.001*	*↓Schirmer ↑IL-1RA*
IL-8	0.2	0.1	N/A	-0.2	0.2	N/A	*0.3*	*0.04*	*↑CFS ↑IL-8*	-0.1	0.4	N/A
EGF	0.1	0.3	N/A	-0.05	0.7	N/A	-0.03	0.8	N/A	-0.06	0.7	N/A
Fractalkine	-0.1	0.5	N/A	-0.001	0.9	N/A	0.01	0.9	N/A	-0.1	0.4	N/A
IL-10	*-0.3*	*0.05*	*↑TER ↓IL-10*	0.2	0.1	N/A	-0.08	0.6	N/A	0.02	0.9	N/A
IL-1 β	*-0.3*	*0.02*	*↑TER ↓IL-1β*	0.1	0.3	N/A	0.1	0.5	N/A	-0.01	0.9	N/A
IL-6	0.03	0.9	N/A	*-0.3*	*0.05*	*↓fTBUT ↑IL-6*	*0.3*	*0.02*	*↑CFS ↑IL-6*	-0.3	0.08	N/A
IP-10	-0.03	0.9	N/A	-0.06	0.7	N/A	-0.2	0.1	N/A	-0.1	0.4	N/A
MCP-1	0.006	0.9	N/A	-0.2	0.3	N/A	0.3	0.03	↑CFS ↑MCP-1	*-0.3*	*0.03*	*↓Schirmer ↑MCP-1*
TNF-α	-0.2	0.3	N/A	-0.1	0.4	N/A	-0.08	0.6	N/A	-0.08	0.6	N/A
VEGF	0.09	0.5	N/A	-0.2	0.1	N/A	0.04	0.8	N/A	0.02	0.9	N/A
Leptin	-0.007	0.9	N/A	0.02	0.9	N/A	0.2	0.2	N/A	-0.04	0.8	N/A
Insulin	0.2	0.09	N/A	0.08	0.6	N/A	0.008	0.9	N/A	0.04	0.8	N/A

Key words: TER, tear evaporation rate; fTBUT, fluorescein tear break-up time; CFS, corneal fluorescein staining; r_s, Spearman correlation coefficient; N/A, not applicable.

Note: Statistically significant differences and their clinical interpretation are reported in *italics*.

Table S3. Relationships of biomarkers and symptoms of DED in the DED-only group. R_s and *p* values are shown, as well as the clinical interpretation of these correlations. A *p* value of ≤0.05 was considered statistically significant. Due to there being a total of 17 subjects in the DED-only group, an r_s of > 0.46 is considered as a positive correlation and < -0.46 is considered as a negative correlation [47, 48].

**Biomarkers**	**DEQS scores (0-100)**			**OSDI scores (0-100)**		
	**r_s**	***p*-value**	**Interpretation**	**r_s**	***p*-value**	**Interpretation**
IL-1RA	0.17	0.52	N/A	0.17	0.51	N/A
IL-8	0.31	0.23	N/A	0.18	0.48	N/A
EGF	0.12	0.64	N/A	0.17	0.5	N/A
Fractalkine	0.38	0.13	N/A	0.28	0.28	N/A
IL-10	0.38	0.14	N/A	0.31	0.23	N/A
IL-1 β	0.2	0.44	N/A	0.02	0.94	N/A
IL-6	-0.13	0.62	N/A	-0.42	0.09	N/A
IP-10	*0.57*	*0.02*	*↑DEQS ↑IP-10*	0.24	0.36	N/A
MCP-1	-0.21	0.41	N/A	0.08	0.76	N/A
TNF-α	*0.49*	*0.04*	*↑DEQS ↑TNF-α*	0.1	0.7	N/A
VEGF	*0.49*	*0.05*	*↑DEQS ↑VEGF*	*0.47*	*0.06*	*↑OSDI ↑VEGF*
Leptin	-0.14	0.58	N/A	-0.46	0.06	N/A
Insulin	0.21	0.41	N/A	0.09	0.74	N/A

Key words: T2D, type 2 diabetes; DED, Dry Eye Disease; r_s, Spearman correlation coefficient; N/A, not applicable.

Note: Statistically significant differences and their clinical interpretation are reported in *italics.*

Table S4. Relationships of biomarkers and symptoms of DED in the T2D+DED group. R_s and *p* values are shown, as well as the clinical interpretation of these relationships. A *p* value of ≤0.05 is considered as statistically significant. Due to there being a total of 47 subjects in the T2D+DED group, an r_s of > 0.27 is considered as a positive relationship and < -0.27 is considered as a negative relationship [47, 48].

**Biomarkers**	**DEQS scores (0-100)**			**OSDI scores (0-100)**		
	**r_s**	***p*-value**	**Interpretation**	**r_s**	***p*-value**	**Interpretation**
IL-1RA	0.08	0.6	N/A	0.02	0.88	N/A
IL-8	0.11	0.47	N/A	0.07	0.66	N/A
EGF	-0.14	0.35	N/A	-0.001	0.99	N/A
Fractalkine	-0.15	0.31	N/A	-0.03	0.84	N/A
IL-10	0.09	0.56	N/A	0.14	0.34	N/A
IL-1 β	0.2	0.17	N/A	0.14	0.34	N/A
IL-6	0.07	0.64	N/A	0.17	0.25	N/A
IP-10	0.13	0.4	N/A	0.09	0.54	N/A
MCP-1	-0.13	0.4	N/A	0.10	0.52	N/A
TNF-α	0.23	0.11	N/A	0.13	0.4	N/A
VEGF	-0.03	0.86	N/A	0.05	0.72	N/A
Leptin	0.22	0.14	N/A	0.21	0.15	N/A
Insulin	0.07	0.62	N/A	0.03	0.86	N/A

Key words: T2D, type 2 diabetes; DED, Dry Eye Disease; r_s, Spearman correlation coefficient; N/A, not applicable.

Table S5. Relationships of biomarkers and clinical data of T2D in the T2D+DED group. R_s and *p* values are shown, as well as the clinical interpretation of these relationships. A *p* value of ≤0.05 is considered as statistically significant. Due to there being a total of 47 subjects in the T2D+DED group, an r_s of > 0.27 is considered as a positive relationship and < -0.27 is considered as a negative relationship [47, 48].

Biomarkers	Duration of T2D			HbA_{1C}			Total Cholesterol			HDL		
	r_s	*p*-value	Interpretation	r_s	*p*-value	Interpretation	r_s	*p*-value	Interpretation	r_s	*p*-value	Interpretation
IL-1RA	-0.16	0.3	N/A	-0.21	0.16	N/A	0.18	0.23	N/A	-0.07	0.64	N/A
IL-8	-0.22	0.13	N/A	0.06	0.68	N/A	-0.03	0.86	N/A	-0.23	0.12	N/A
EGF	-0.19	0.19	N/A	-0.13	0.37	N/A	0.06	0.7	N/A	-0.005	0.97	N/A
Fractalkine	-0.12	0.44	N/A	-0.08	0.6	N/A	0.24	0.1	N/A	0.03	0.86	N/A
IL-10	0.17	0.27	N/A	0.02	0.89	N/A	-0.09	0.54	N/A	-0.17	0.27	N/A
IL-1 β	0.06	0.68	N/A	0.11	0.45	N/A	-0.24	0.11	N/A	-0.07	0.63	N/A
IL-6	-0.05	0.75	N/A	0.04	0.81	N/A	0.06	0.71	N/A	-0.04	0.8	N/A
IP-10	-0.08	0.62	N/A	0.005	0.97	N/A	0.12	0.44	N/A	-0.18	0.23	N/A
MCP-1	-0.03	0.86	N/A	-0.02	0.92	N/A	-0.26	0.08	N/A	-0.21	0.15	N/A
TNF-α	-0.11	0.46	N/A	0.21	0.15	N/A	-0.002	0.99	N/A	-0.17	0.26	N/A
VEGF	*-0.3*	*0.04*	*↑Duration of T2D ↓VEGF*	0.1	0.5	N/A	0.19	0.19	N/A	0.009	0.95	N/A
Leptin	0.12	0.41	N/A	0.01	0.95	N/A	-0.14	0.34	N/A	-0.23	0.11	N/A
Insulin	*-0.31*	*0.03*	*↑Duration of T2D ↓Insulin*	-0.19	0.21	N/A	-0.03	0.87	N/A	*-0.39*	*0.007*	*↑HDL ↓Insulin*

Key words: T2D, type 2 diabetes; HbA_{1C}, glycated haemoglobin; HDL, high density lipoprotein; r_s, Spearman correlation coefficient; N/A, not applicable.
Note: Statistically significant differences and their clinical interpretation are reported in *italics*.
